# Breast implant-associated anaplastic large cell lymphoma: a pictorial review

**DOI:** 10.1007/s13244-018-0652-z

**Published:** 2018-09-04

**Authors:** Amit Chacko, Thomas Lloyd

**Affiliations:** Department of Diagnostic Radiology, Princess Alexandra Hospital, 199 Ipswich Rd, Woolloongabba, Brisbane, Queensland 4102 Australia

**Keywords:** Breast imaging, Oncologic imaging, Ultrasound, Nuclear imaging, Lymph

## Abstract

**Abstract:**

Breast implant-associated anaplastic large cell lymphoma (BIA-ALCL) is a newly described and rare T-cell lymphoma of the breast. Since 2007, there have been 56 cases of confirmed BIA-ALCL in Australia and New Zealand. The incidence is believed to be on the rise as the prevalence of elective breast implantation increases. In 2016, the World Health Organization (WHO) classified BIA-ALCL as a recognised entity and emphasised the importance of surgical management of the disease. BIA-ALCL typically presents as a delayed, non-infective fluid collection around a textured breast implant or residual fibrous scar capsule. The mean age of presentation is 47 years, with an average time frame of 7.5 years following breast implantation. Although rare, BIA-ALCL is increasing in incidence. To avoid delays in diagnosis, radiologists should consider this form of lymphoma in the differential of any non-acute peri- or post-prosthetic effusion, and suggest cytological evaluation, so as not to miss this rare but important diagnosis.

**Teaching Points:**

• *BIA-ALCL is a newly described and rare T-cell lymphoma of the breast.*

• *Since 2007, there have been 56 cases of confirmed BIA-ALCL in Australia and New Zealand.*

• *BIA-ALCL presents as a delayed, non-infective fluid collection.*

• *The effusion typically accumulates around a textured breast implant or residual fibrous capsule.*

## Background

Breast implant-associated anaplastic large cell lymphoma (BIA-ALCL) is a newly described and rare primary T-cell lymphoma of the breast. Since 2007, there have been 56 cases of confirmed BIA-ALCL in Australia and New Zealand [1, 2]. The incidence is believed to be on the rise as the prevalence of elective breast implantation increases [3]. In 2016, the World Health Organization (WHO) classified BIA-ALCL as a recognised entity and emphasised the importance of surgical management of the disease [4].

Non-Hodgkin lymphomas (NHLs) are haematological malignancies that rarely involve the breast. NHLs that involve the breast account for less than 1% of breast cancers and are predominantly B-cell in origin [5]. BIA-ALCLs are CD30 T-cell-positive derived lymphomas from the NHLs group [1]. They account for only 3% of NHLs. The exact pathophysiology of BIA-ALCL is unclear, but there is growing evidence that biofilm surrounding the implant stimulates lymphocyte production, which triggers a cycle of inflammation that ultimately results in BIA-ALCL [6].

The following cases are from a selection of patients with confirmed BIA-ALCL, with discussion of the presentation, imaging modalities and staging of the disease process. Our tertiary centre has treated eight confirmed cases of BIA-ALCL.

## Case 1

**Fig. 1:**
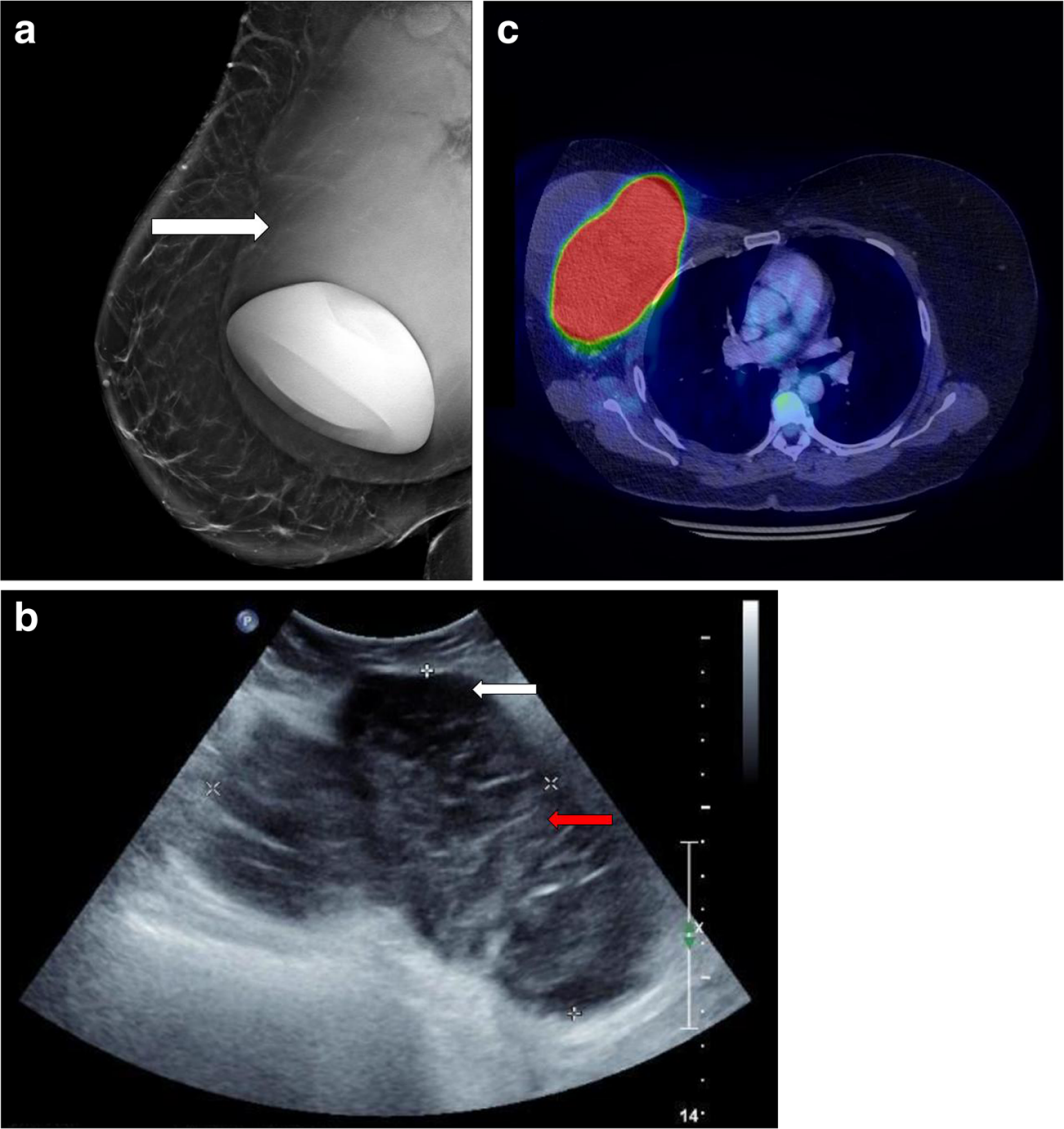
**a** The mammogram revealed that the implant was displaced anteriorly and inferiorly by a large, lobular, ill defined, soft tissue density mass (*white arrow*). The implant appears intact but compressed.** b** Ultrasound revealed a peri-implant effusion (*white arrow*), with the implant displaced and compressed by a large lobular solid heterogeneous mass (*red arrow*). Masses, as in this case, are unusual in breast implant-associated anaplastic large cell lymphoma (BIA-ALCL). The diagnosis was confirmed by core biopsy of the mass.** c** Positron emission tomography/computed tomography (PET/CT) revealed a large mixed-density mass with intense FDG activity, deep within and invading the right breast and pectoralis muscles. There was metastatic disease spread to the lung and bone

Patient A is a 48-year-old female referred for investigation of progressive swelling of her right breast. The patient previously had left-sided breast cancer, for which she underwent a total mastectomy. Subsequently, she underwent breast implantation for cosmetic purposes. She was referred for a mammogram (Fig. 1a). Mammograms are typically used in conventional breast cancer screening but cannot accurately distinguish between an effusion and a mass [7].

Patients with BIA-ALCL often present to their primary clinician with breast enlargement, asymmetry, skin rash, contracture or lymphadenopathy [8]. The average time frame of presentation is 7 years following breast implantation [1]. Initial presentation often manifests as a peri-prosthetic effusion surrounding an implant on ultrasound. Any new effusion around an implant of more than 12 months of age should prompt consideration of BIA-ALCL. Patient A subsequently underwent ultrasound assessment (Fig. 1b).

The most notable abnormality of BIA-ALCL is an effusion in relation to the breast implant [7]. These can be peri-prosthetic or even present in the subcutaneous layer [9]. Aspirated fluid must be sent for flow cytometry and not simply for microscopy and culture, with the pathologist alerted to the possibility of the BIA-ALCL. If ultrasound examination is indeterminate, then magnetic resonance imaging (MRI) or positron emission tomography/computed tomography (PET/CT) should be considered for further evaluation (Fig. 1c). The patient was subsequently admitted for implant removal with capsulectomy and adjuvant chemotherapy.

## Case 2

**Fig. 2:**
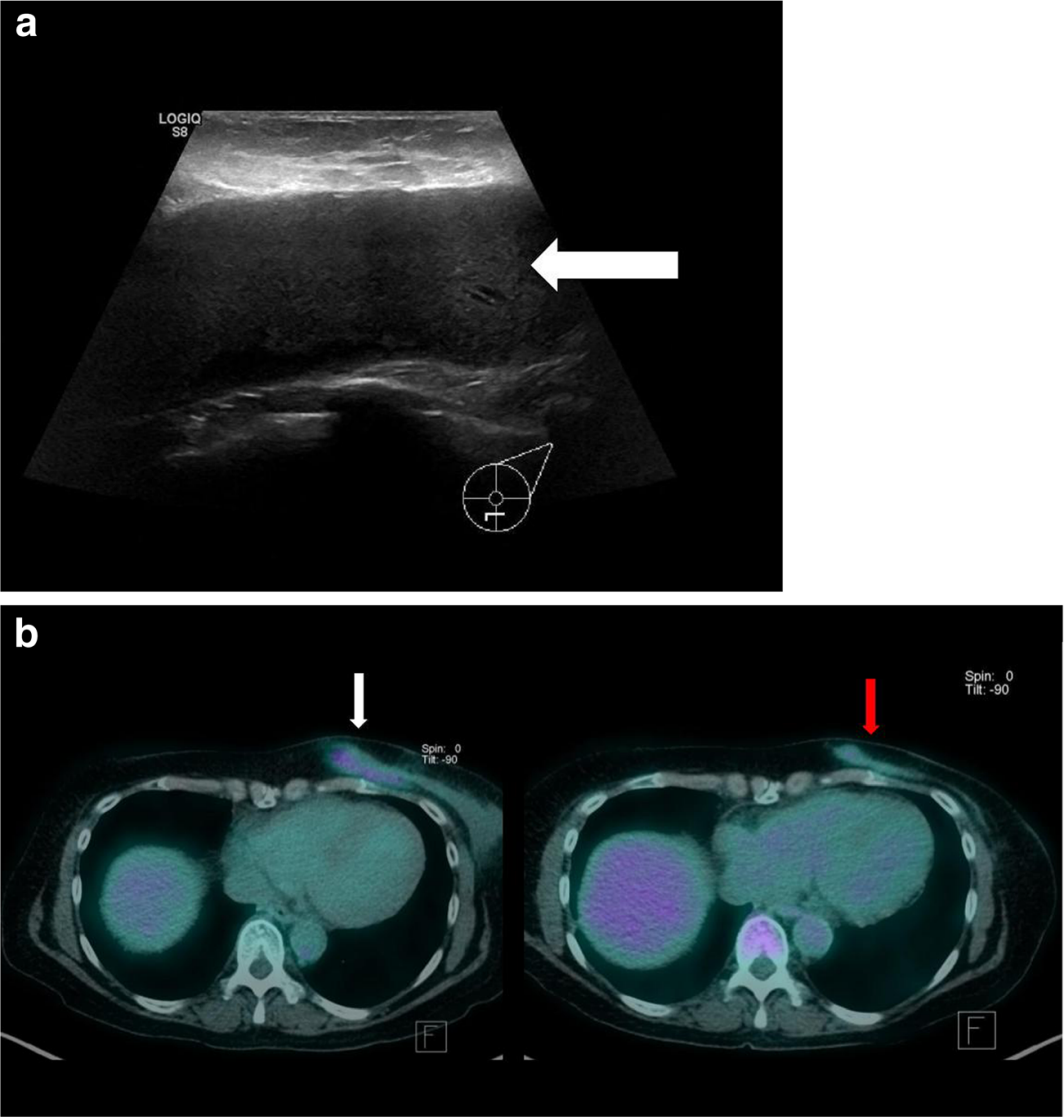
**a** Ultrasound revealed a large effusion with no signs of infection (*white arrow*). Fortunately, the aspirated fluid was sent for cytology, which confirmed BIA-ALCL.** b** PET/CT revealed a flattened rim of soft tissue, located inferomedially in the left breast, with ill-defined margins and moderate FDG uptake (*white arrow*). The patient subsequently received six cycles of chemotherapy and targeted radiotherapy. Restaging PET/CT revealed complete metabolic response (*red arrow*)

Patient B is a 64-year-old female with bilateral breast implants who presented to her GP with a painful left breast. Turbid fluid was aspirated inferior to the left breast prosthesis. It was concluded that the implant was infected and the implants were removed. Unfortunately, the aspirated fluid was not sent to pathology for assessment. The patient did not undergo a capsulectomy. She represented to her GP 2 years later with unilateral left breast swelling and underwent ultrasound assessment (Fig. 2a). This case highlights that BIA-ALCL can even occur from a residual fibrous capsule.

Patient B was referred for a staging PET/CT (Fig. 2b). Evaluation with PET can vary from diffuse [10–12] to focal [13, 14] FDG uptake surrounding the implant or its capsule. FDG uptake can also appear in regional lymph nodes, suggestive of metastatic progression [10, 14–16].

## Case 3

**Fig. 3:**
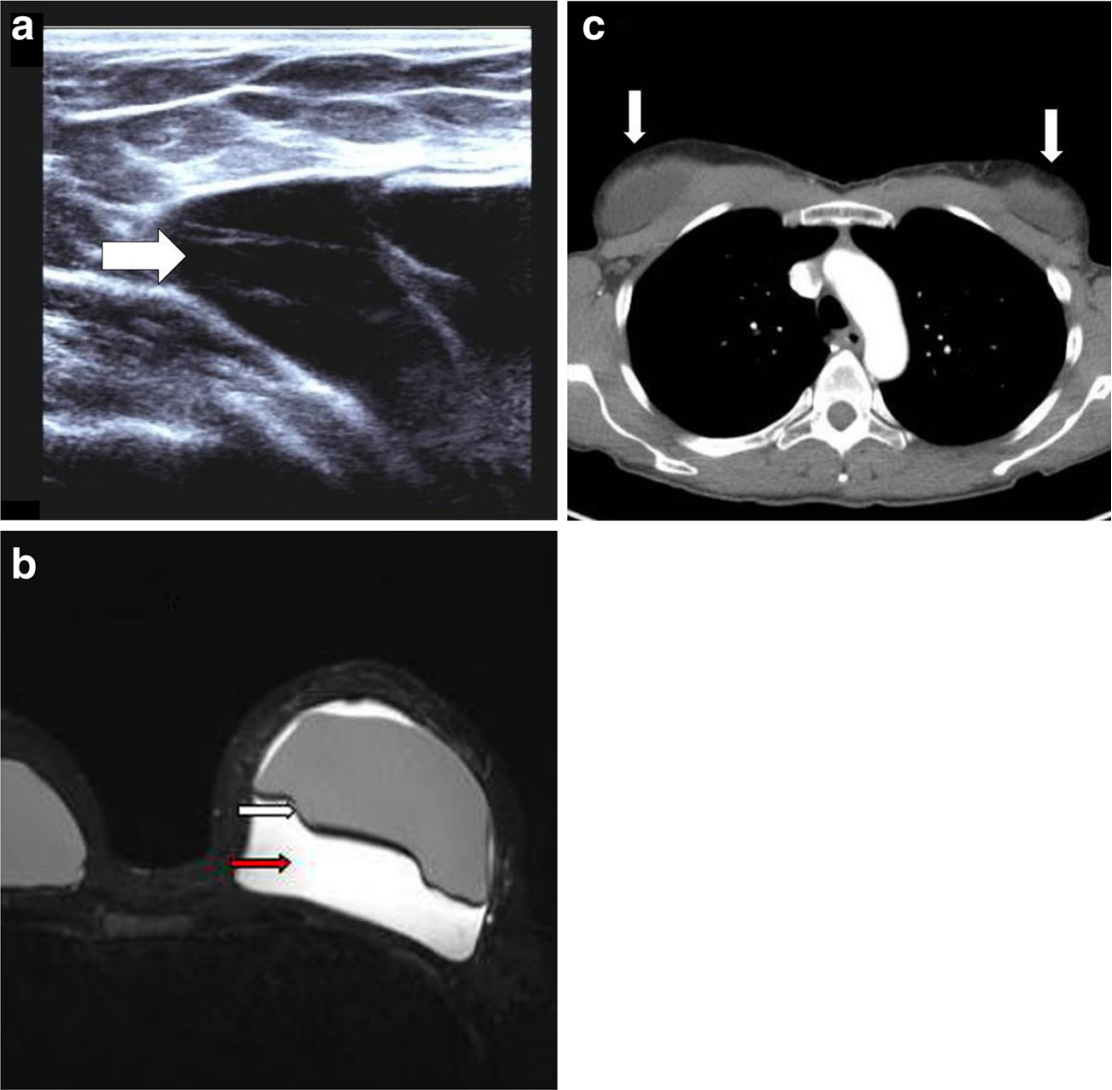
**a** Ultrasound revealed a large septated seroma (*white arrow*), which was aspirated the following day. Cytology confirmed BIA-ALCL. Ultrasound has a sensitivity of 84% and specificity of 75% for detecting an effusion. These figures are similar or better than CT or magnetic resonance imaging (MRI) in effusion detection [7].** b** MRI provides characterisation of the implant’s capsule, defining enhancement and thickening [14, 15]. This makes it the modality of choice for defining the implant capsule (*white arrow*) [7]. BIA-ALCL typically presents as a delayed, non-infective fluid collection surrounding the implant (*red arrow*) or its surrounding scar capsule, with or without evidence of capsular rupture [13].** c** Staging CT revealed a small to moderate effusion adjacent to both breast implants (*white arrows*)

Patient C is 33-year-old female who presented to her cosmetic surgeon with a sudden and rapid increase in the size of her left breast. The patient had bilateral textured breast implants inserted 4 years previously. The patient was referred for ultrasound assessment (Fig. 3a).

The patient underwent MRI assessment (Fig. 3b). The external structure of the implant has been found to statistically influence the risk of developing BIA-ALCL, with the majority of cases occurring with textured breast implants [13]. There has been no significant difference in incidence between saline and silicone implants. There is also inadequate evidence to comment if implant location plays a role in developing BIA-ALCL [5].

The patient was staged with CT (Fig. 3c). Many patients with BIA-ALCL have an effusion, mass or lymphadenopathy on CT evaluation [14]. Other findings can include irregularity of implant contour and capsular thickening [10, 14, 15]. The patient underwent bilateral implant removal, with bilateral capsulectomies. Subsequent PET/CT showed complete metabolic remission. Surprisingly, the patient had bilateral breast implantations the following year, despite being warned of the risk of BIA-ALCL recurrence. The patient is being closely monitored for evidence of relapse.

## Conclusion

Although rare, breast implant-associated anaplastic large cell lymphoma (BIA-ALCL) is increasing in incidence, and commonly manifests as an effusion around a textured breast implant or residual fibrous capsule. To avoid delays in diagnosis, radiologists should consider this form of lymphoma in the differential of any peri- or post-prosthetic effusion.
